# Book Reviews

**Published:** 1898-01

**Authors:** 


International Clinics. A Quarterly of Clinical Lectures on Medicine, Neurology, Surgery, Gynecology, Obstetrics, Ophthalmology, Laryngology, Pharyngology, Rhinology, Otology and Dermatology, and specially prepared articles on treatment. By professors and lecturers in the leading medical colleges of the United States, Germany, Austria, France, Great Britain and Canada. Edited by Judson Daland, M. D., (Univ. of Penna.) Philadelphia, Instructor in Clinical Medicine and Lecturer on Physical Diagnosis in the University of Pennsylvania ; Assistant Physician to the Hospital of the University of Pennsylvania, etc.; Fellow of the College of Physicians of Philadelphia. J. Mitchell Bruce, M. D., F. R. C. P., London, England, Physician to, and Lecturer on, the Principles and Practice of Medicine at the Charing Cross Hospital. David W. Finlay, M. D., F. R. C. P., Aberdeen, Scotland, Professor of Practice of Medicine in the University of Aberdeen ; Physician to, and Lecturer on, Clinical Medicine in the Aberdeen Royal Infirmary, etc. Volume III. Seventh series. 1897. Octavo, pp. xii.—360. Philadelphia : J. B. Lippincott Co. 1897.

In this volume there is one lecture under the general head of Drugs and remedial agents, eight lectures under Treatment, ten lectures under Medicine, two lectures under Neurology, seven lectures under Surgery, five lectures under Gynecology and obstetrics, one lecture under Ophthalmology, two lectures under Laryngology, pharyngology, rhinology and otology, one lecture under Dermatology—a total of thirty-seven discourses that are very well distributed among the important departments of medicine and surgery. The authors are for the most part men of considerable experience and some of them are of conspicuous eminence. Dr. E. L. Shurley, of Detroit, speaks interestingly as well as instructively, especially on the treatment of phthisis pulmonalis. This author has great experience in the diseases of the upper-air tract, being an accomplished laryngologist as well as skilful in internal medicine. What he says, therefore, on these subjects is entitled to great credence.

An especially interesting discourse is that on the surgical treatment of gall-stones by Prof. James F. W. Ross, of Toronto. No one is more competent to treat this subject scientifically than Dr. Ross. His lecture is illustrated by four schematic drawings in colors and a plate giving specimens of various gall-stones removed by operation.

A lecture by Dr. A. H. Freeland Barbour, of Edinburgh, on Bleeding in pregnancy and labor is of special interest and it is illustrated by six drawings.

A lecture by Prof. J. McFadden Gaston, of Atlanta, on Hematoma, also illustrated with a plate showing hematoma near the first lumbar vertebra following strain, deserves special mention. A number of other lectures are to be commended and the entire volume merits praise.

A Manual of Legal Medicine for the Use of Practitioners and Students of Medicine and Law. By Justin Herold, A. M., M. D., formerly Coroner's Physician of New York City and County, etc., etc. Octavo, pp. xv.—678. Philadelphia: J. B. Lippincott Co. 1898.

The author of this book states that he has prepared it not only to embrace the contents of the more elaborate and classic works in a book of medium size, but to present them together with original material condensed into a comparatively small but not too scant space. It is a recognised fact that busy physicians cannot always find time to familiarise themselves with the facts and theories of encyclopedic works; such, however, will find this treatise an excellent one to consult. It is written by a man of practical experience in the conduct of medico-legal cases.

The work is divided into two parts: the first being devoted to the consideration of toxicology, to which sixteen chapters have been assigned, and the second part to forensic medicine, including pharmaceutical jurisprudence, to which twenty-six chapters are appropriated. In one chapter the duties and powers of coroners are considered. While the office of coroner is of ancient origin, it is one that nowadays seems ill-adapted to our necessities and environment. Herold makes reference in the same chapter to expert witnesses. It seems to us that it would be a good plan, now that public attention is turned toward reforms in these matters, to abolish coroners and expert witnesses together, as both have brought reproach to the profession of medicine. Instead, let us have official medical examiners, according to the fashion in Massachusetts, to take the place of coroners, and commissions appointed by the court to take the place of expert witnesses.

One of the chapters is devoted to the consideration of life insurance, which will be found of interest to every physician. It would be the work of supererogation to review this book, chapter by chapter, but it is proper for us to affirm that it will be found helpful to every physician who has to do with medico-legal cases, and to both medical and law students. Especially do we commend it to such as have neither time, inclination nor means to obtain and study the larger works on medical jurisprudence.

Lectures on the Malarial Fevers. By William Sidney Thayer, M. D., Associate Professor of Medicine in the Johns Hopkins University. Small octavo, pp. vi.—326. New York: D. Appleton & Co. 1897.

In this treatise, which is a grouping of nine lectures delivered at Johns Hopkins University, the author has outlined the present scientific knowledge of which we are in possession concerning the malarial fevers. It is based on the parasitic origin of these fevers according to the discovery of Laveran, made in Algeria in 1880, and published in the *Bulletin* of the Academy of Medicine of Paris on the 23d of November in the same year.

Thayer draws attention at the outset to the improper use of the term "malaria" so common on the lips of the laity. Scientific men understand that there is no disease named malaria; nevertheless, they frequently permit themselves to apply the word in that sense, hence the laity have readily fallen into an error that it will be difficult to correct. It is quite proper, as Thayer points out, to use the term "the malarial fevers," by which is meant a class of fevers due to a specific microörganism which yields to treatment by quinine.

Thayer traces the disease through the various blood changes that it causes, gives a clinical description of the maladies in question, describes their sequelæ and complications, their morbid anatomy and general pathology, devoting his last lecture to diagnosis, prognosis, treatment and prophylaxis. The text is supported by nineteen illustrative charts, showing the temperature curves in the several forms of these fevers and three plates depicting the parasites that belong to each class.

No one can fail to appreciate the value of this work who will read it; for it is the most distinct contribution to the literature of the subject that has yet appeared and must necessarily take high rank in scientific medicine.

---

A Text-Book of the Practice of Medicine. By James M. Anders, M. D., Ph. D., LL. D., Professor of the Practice of Medicine and of Clinical Medicine in the Medico-Chirurgical College, Philadelphia; Attending Physician to the Medico-Chirurgical and Samaritan Hospitals, Philadelphia, etc. Octavo, pp. 1287. Illustrated. Philadelphia: W. B. Saunders, 925 Walnut street. 1896.

A new treatise on internal medicine must present some essential reason for its appearance at this time in order to attract the eye of the profession. The tendency of practical physicians is toward the purchase of monographs. Text-books, therefore, must be written mainly in the interest of students. It must be confessed that this author, being a practical teacher, has presented his book in a form that will make it especially attractive to the student. Take, for instance, the section (the book is not divided into chapters in the ordinary way) on diphtheria, which is considered under the following heads: definition, pathology, etiology, immunity, symptoms, complications, diagnosis, prognosis and treatment. Each of these also have subheads, of which the following is an example: general head, etiology; subheads, bacteriology, cite of infection, the toxins, associated microbes, modes of infection and predisposing factors. It will be readily understood how easily accessible a particular subject becomes in a treatise classified in this manner.

Anders has paid special attention to the clinical pictures of disease, though he has not neglected pathology. In considering the etiology of most affections, he has given prominence to bacteriological phenomena. Another interesting feature of this treatise is the fact that in many instances differential diagnosis has been tabulated, there being no less than fifty-six diagnostic tables scattered throughout the work, most of which are original. The author is to be congratulated upon the adoption of modern orthography and terminology, which is in accordance with the judgment and practice of modern lexicographers. It is difficult to understand how authors and editors still cling to diphthongs and other ancient methods of spelling in spite of the best modern authority on philology.

In such a work it would have been in order to have illustrated more profusely; nevertheless, the pictures published are of excellent quality and helpful in character. The paper is not heavy, but is well finished, and the presswork is admirable. We have no doubt this treatise will become a text-book in many, if not the majority, of our medical colleges.

Index Catalogue of the Library of the Surgeon-General's Office United States Army. Authors and subjects. Second series. Vol. II. B. Bywater. Washington: Government Printing Office. 1897.

This book, which is the second volume of the second series of this great catalogue, contains 15,732 author-titles, representing 6,383 volumes and 14,802 pamphlets. It also contains 5,774 subject-titles of separate books and pamphlets and 21,725 titles of articles in periodicals. It is prepared under the direction of the surgeon-general by Col. D. L. Huntington, M. D., deputy surgeon-general, U. S. Army, at Washington.

---

The Essentials of Obstetrics. By Charles Jewett, M. D., Professor of Obstetrics in the Long Island College Hospital, Brooklyn, New York. Duodecimo volume of 356 pages, with seventy-eight illustrations and three colored plates. New York and Philadelphia: Lea Brothers & Company. 1897.

The purpose of this work is modestly stated by the author in the following words: "If these pages shall be found useful as an introduction to the more elaborate treatise, and as a guide in following the didactic and the practical teaching of the college course, the author's object will have been gained."

Dr. Jewett is an experienced teacher and well knows the needs of the student. He has succeeded in producing a practical book that is concise, yet clear in its descriptions, elementary in character and helpful in form. When a student begins the study of obstetrics he cannot do better than to adopt this book as a guide.

---

The Johns Hopkins Hospital Reports. Volume VI. Baltimore: The Johns Hopkins Press. 1897.

This excellent report, containing 414 pages, is devoted to several different departments of the hospital and shows what careful, painstaking work is done by the heads of the various departments. The volume contains a masterful study of the lesions produced by the action of certain poisons on the cortical nerve cell by Henry J. Berkley, illustrated with some of the most beautiful photomicrographs of nerve cells that the reviewer has ever seen.

The report in pathology embraces several sub-reports by Drs. Thomas S. Cullen, G. P. Wilkins, William D. Barker and Simon Flexner, as Fatal puerperal sepsis due to introduction of an elm tent; Pregnancy in a rudimentary uterine horn, rupture, death; Probable migration of ovum and spermatozoa; Adeno-myoma uteri diffusum benignum; A bacteriological and anatomical study

